# Nano-Hydroxyapatite-Based Mouthwash for Comprehensive Oral Care: Activity Against Bacterial and Fungal Pathogens with Antioxidant and Anti-Inflammatory Action

**DOI:** 10.3390/ma18153567

**Published:** 2025-07-30

**Authors:** Tomasz M. Karpiński, Magdalena Paczkowska-Walendowska, Judyta Cielecka-Piontek

**Affiliations:** 1 Chair and Department of Medical Microbiology, Poznan University of Medical Sciences, Rokietnicka 10, 60-806 Poznan, Poland; tkarpin@ump.edu.pl; 2 Department of Pharmacognosy and Biomaterials, Poznan University of Medical Sciences, 60-806 Poznan, Poland; jpiontek@ump.edu.pl; 3 Science-Bridge Sp. z o.o., Chociszewskiego 24/8, 60-258 Poznan, Poland

**Keywords:** nano-hydroxyapatite, *Staphylococcus aureus*, biofilm, mouthwash, antibacterial activity, dental implants, cetylpyridinium chloride, antioxidant, anti-inflammatory, oral microbiology

## Abstract

**Background/Objectives:** The growing demand for biocompatible and fluoride-free alternatives in oral care has led to the development of formulations containing nano-hydroxyapatite (nanoHAP). This study aimed to evaluate the antimicrobial, antibiofilm, antioxidant, and anti-inflammatory properties of a novel mouthwash containing nanoHAP, zinc lactate, D-panthenol, licorice extract, and cetylpyridinium chloride, with particular focus on its efficacy against *Staphylococcus aureus* and its biofilm on various dental materials. **Methods:** The antimicrobial activities of the mouthwash KWT0000 and control product ELM were assessed via minimal inhibitory concentration (MIC) testing against selected Gram-positive and Gram-negative bacteria and *Candida* fungi. Antibiofilm activity was evaluated using fluorescence and digital microscopy following 1-h exposure to biofilms of *Staphylococcus aureus*, *Pseudomonas aeruginosa*, and *Candida albicans*. The efficacy was compared across multiple dental materials, including titanium, zirconia, and PMMA. Antioxidant capacity was determined using the 2,2-diphenyl-1-picrylhydrazyl radical (DPPH) assay, and anti-inflammatory potential via hyaluronidase inhibition. **Results:** KWT0000 exhibited strong antimicrobial activity against *S. aureus* and *C. albicans* (MICs: 0.2–1.6%) and moderate activity against Gram-negative strains. Fluorescence imaging revealed significant biofilm disruption and bacterial death after 1 h. On metallic surfaces, especially polished titanium and zirconia, KWT0000 reduced *S. aureus* biofilm density considerably. The formulation also demonstrated superior antioxidant (55.33 ± 3.34%) and anti-inflammatory (23.33 ± 3.67%) activity compared to a fluoride-based comparator. **Conclusions:** The tested nanoHAP-based mouthwash shows promising potential in antimicrobial and antibiofilm oral care, particularly for patients with dental implants. Its multifunctional effects may support not only plaque control but also soft tissue health.

## 1. Introduction

Contemporary dentistry increasingly adopts solutions based on biomimetics—substances that mimic the natural components of tooth and periodontal tissue [1]. One promising direction is the use of synthetic nano-hydroxyapatite (nanoHAP), the main inorganic constituent of enamel and dentin. Due to its biocompatibility, ability to remineralize tissues, and many other clinically significant features, it may serve as an effective alternative to fluoride in the prevention and treatment of oral diseases [2].

In this study, a specially formulated mouthwash containing nano-hydroxyapatite, zinc lactate, D-panthenol, licorice extract, and 0.05% cetylpyridinium chloride was used. This composition offers potential remineralizing [3,4], anti-inflammatory, and antibacterial properties [5,6,7,8,9,10,11]. Despite its well-documented effectiveness in inhibiting the caries process, fluoride shows limited activity in implantological environments, where natural enamel susceptible to demineralization is absent.

In this context, properties that reduce bacterial biofilm adhesion and provide ongoing anti-inflammatory effects become significantly more important—particularly due to the susceptibility of peri-implant tissues to inflammation, which may lead to complications such as peri-implantitis [12]. In the case of natural teeth, however, it is worth noting that the fluoride concentration in many commercially available mouthwashes (e.g., 250 ppm F^−^), is too low to demonstrate clinically significant anti-caries effects [13]. Clinical studies have consistently shown that hydroxyapatite toothpaste is not inferior to fluoride-containing toothpaste in preventing dental caries across different age groups. Evidence from randomized controlled trials in children [14], adolescents undergoing orthodontic treatment [15], and adults [16] confirms the comparable efficacy of hydroxyapatite to fluoride toothpastes containing up to 1450 ppm fluoride. Due to the risk of ingestion, higher doses cannot be used, making hydroxyapatite a safe and effective alternative [16]. The pH buffering action and protective calcium- and phosphate-releasing characteristics of hydroxyapatite in cariogenic biofilms are significant benefits of its use in caries prevention [17].

The scientific rationale for combining these agents is to create a multifunctional mouthwash that addresses multiple aspects of oral health simultaneously, namely antimicrobial protection, anti-inflammatory support, mucosal regeneration, and implant surface compatibility. This is particularly relevant for patients with dental implants, where biofilm control must be achieved without compromising peri-implant tissue health.

The aim of our study was to compare the effectiveness of mouthwashes containing nano-hydroxyapatite and fluoride in the form of amine fluoride in terms of their activity against selected bacteria and fungi (*Streptococcus mutans*, *Staphylococcus aureus*, *Escherichia coli*, *Klebsiella pneumoniae*, *Acinetobacter baumannii*, *Pseudomonas aeruginosa*, and *Candida albicans*, *Candida auris*) and their ability to reduce bacterial adhesion to the surfaces of commonly used implant materials: titanium and zirconium oxide with various coatings. In this context, a key element was the evaluation of biofilm adhesion—both quantitatively and qualitatively—to these materials under conditions approximating the oral environment, and also their antioxidant and anti-inflammatory activity supporting daily oral hygiene.

The research presented here constituted an important stage in the development of the Harmony Care mouthwash—a modern oral hygiene product containing nano-hydroxyapatite and ingredients that support antibacterial and regenerative actions. The results obtained serve as a basis for further analyses and expansion of clinical applications of the preparation, both in patients with natural dentition and those with prosthetic restorations or implants.

## 2. Materials and Methods

### 2.1. Material

Two mouthwashes were used in this study. The first Harmony Care product, manufactured by Get Harmony Lab, Poznan, Poland, referred to as KWT0000, is a mouthwash containing hydroxyapatite, licorice extract, D-panthenol, zinc lactate, and 0.05% cetylpyridinium chloride. The second product (Elmex^®^ manufactured by Colgate-Palmolive Services Sp. z o.o., Warsaw, Poland), referred to as ELM, used as a comparative mouthwash, contains Olaflur (250 ppm), sodium fluoride (250 ppm), and sodium salt of anise acid.

### 2.2. Microbiological Activity

#### 2.2.1. Minimal Inhibitory Concentrations (MIC)

To determine the minimal inhibitory concentrations (MIC), the microdilution method was used. Cultures were grown in tryptic soy broth (Graso Biotech, Starogard Gdański, Poland) on 96-well plates (Nest Scientific Biotechnology, Wuxi, Jiangsu, China), with a final volume of 100 µL per well. Serial dilutions of mouthwashes were prepared, starting from 100% to 0.1%. A detailed description of the methodology can be found in our previous publication [18]. The study utilized clinical strains of Gram-positive bacteria, *Streptococcus mutans* and *Staphylococcus aureus*, Gram-negative bacteria *Acinetobacter baumannii*, *Escherichia coli*, *Klebsiella pneumoniae*, and *Pseudomonas aeruginosa*, and yeast-like fungi *Candida albicans* and *C. auris*, from the portfolio of the Chair and Department of Medical Microbiology, Poznań University of Medical Sciences. For 24 to 48 h, the plates were incubated at 36 °C. Following incubation, 10 µL of a 1% aqueous solution of 2,3,5-triphenyl tetrazolium chloride (TTC) (Sigma Aldrich, Poznań, Poland) was added, and MIC values were assessed visually or, alternatively, by a color reaction.

#### 2.2.2. Anti-Biofilm Activity

The anti-biofilm activity of mouthwashes was evaluated using fluorescence microscopy combined with the LIVE/DEAD BacLight Bacterial Viability Kit (Life Technologies, Carlsbad, CA, USA), following a previously established protocol [19]. The study was against *S. aureus*, *P. aeruginosa*, and *C. albicans*. Biofilms were cultivated in 12-well plates (Nest Scientific Biotechnology, Wuxi, China) containing tryptic soy broth (TSB) and incubated at 37 °C for 48 h. After incubation, the culture medium was discarded, and the wells were gently rinsed with saline. A mixture of 3 µL of the viability dyes in 1 mL of saline was then added to each well, according to the manufacturer’s guidelines. The plates were incubated in the dark at room temperature for 15 min. Subsequently, the stained biofilms were examined using a fluorescence microscope (Leica DM1000 LED, Leica Microsystems GmbH, Wetzlar, Germany), and images were acquired with a Progres Gryphax camera (Jenoptik AG, Jena, Germany). Additionally, the activity of mouthwashes against *Staphylococcus aureus* was analyzed using a Keyence VHX-S770E digital microscope (Keyence International, Mechelen, Belgium) on some dental devices in the form of discs with a diameter of 1 cm: poly (methyl methacrylate) (PMMA) (Cad/Cam Disc with Shoulder Multilayer, Polident d.o.o., Volcja Draga, Slovenia), resin A1 (optiprint lumina, dentona AG, Otto-Hahn-Straße 27, DE-44227 Dortmund, Germany), anodized/polished titanium (Magnum Hyperone—dental titanium alloy for CAD/CAM milling, type 4, Mesa Italia, via dell’Artigianto 35/37, 25039 Travagliato, Italy), monolithic zirconia (Zirconium, Dental Zirconia Ceramics, Shenzhen Upcera Dental Technology Co., Ltd., Shenzhen, China, manufactured for Mikran.pl), zirconia with glaze (Zirconium, Dental Zirconia Ceramics, Shenzhen Upcera Dental Technology Co., Ltd., manufactured for Mikran.pl + Glaze Miyo: InSync glaze paste fluor, Chemichl AG, Landstrasse 114, FL-9490 Vaduz, Liechtenstein), and polished zirconia (Zirconium, Dental Zirconia Ceramics, Shenzhen Upcera Dental Technology Co., Ltd., manufactured for Mikran.pl + Polishing Paste: Zirkopol, Diamant-Polierpaste, feguramen, 74722 Buchen, Germany).

### 2.3. Antioxidant Properties

The antioxidant properties were assessed using the 2,2-diphenyl-1-picrylhydrazyl (DPPH) radical scavenging assay, following a method previously reported [20]. In short, 25 μL of mouthwash was mixed with 175 μL of a DPPH solution (0.2 mmol/L) and incubated for 30 min in the dark at 25 °C. Subsequently, the absorbance was recorded at 517 nm against a blank (25 μL of water mixed with 175 μL of methanol) using a Multiskan GO 1510 microplate reader (Thermo Fisher Scientific, Vantaa, Finland).

### 2.4. Anti-Inflammatory Properties

The inhibition of hyaluronidase was evaluated using a turbidimetric assay, as outlined previously [20]. In brief, 25.0 µL of incubation buffer, 25.0 µL of hyaluronidase solution (30 U/mL), 10.0 µL of the mouthwash, and 15.0 µL of acetate buffer were combined in a microplate well. After incubation at 37 °C for 15 min with gentle shaking, 25.0 µL of a hyaluronic acid (HA) solution was added. The mixture was incubated for an additional 45 min under the same conditions. Following incubation, 200.0 µL of cetyltrimethylammonium bromide (CTAB) solution in 2% sodium hydroxide was added. After a 10-min incubation at 25 °C without agitation, absorbance was measured at 600 nm using the Multiskan GO 1510 microplate reader (Thermo Fisher Scientific, Vantaa, Finland).

### 2.5. Statistical Analysis

The statistical analysis was carried out using Statistica 13.3. The ANOVA test and Tukey’s post hoc range test for multiple comparisons were used to examine the variances between the mean values and Duncan’s post hoc test. At *p* < 0.05 differences across groups were deemed significant.

## 3. Results and Discussion

The oral cavity is a complex ecosystem colonized by hundreds of microbial species, many of which are involved in the development of local diseases such as dental caries, gingivitis, and periodontitis. Moreover, microorganisms residing in the mouth can enter the bloodstream, particularly in immunocompromised individuals, posing a risk for serious systemic infections such as endocarditis, pneumonia, or sepsis.

This study includes a diverse panel of clinically relevant pathogens. Gram-positive bacteria such as *Streptococcus mutans*, a primary agent of dental caries [21], and *Staphylococcus aureus*, which is associated with soft tissue infections and healthcare-associated infections [22], are evaluated. Among Gram-negative bacteria, *Acinetobacter baumannii, Escherichia coli, Klebsiella pneumoniae*, and *Pseudomonas aeruginosa* are included due to their opportunistic nature and frequent multidrug resistance. These pathogens are known to colonize mucosal surfaces, including the oral cavity, and can lead to severe infections, particularly in hospital settings. Notably, the presence of certain subgingival non-oral species such as *A. baumannii* and *P. aeruginosa* has been associated with a higher percentage of periodontal sites exhibiting suppuration on probing, greater clinical attachment loss, and more aggressive forms of periodontitis, highlighting their potential role in exacerbating oral diseases [23]. Additionally, the study examines the antifungal potential of mouthwashes against *Candida albicans*, the most common cause of oral candidiasis, and *Candida auris*, an emerging, multidrug-resistant fungus with significant epidemiological importance.

In an era of increasing antimicrobial resistance, the role of effective preventive strategies becomes more critical. Mouthwashes, beyond their cosmetic and breath-freshening roles, may serve as preventive tools that help reduce the microbial load in the oral cavity and limit the colonization by pathogenic organisms.

**Figure 1:**
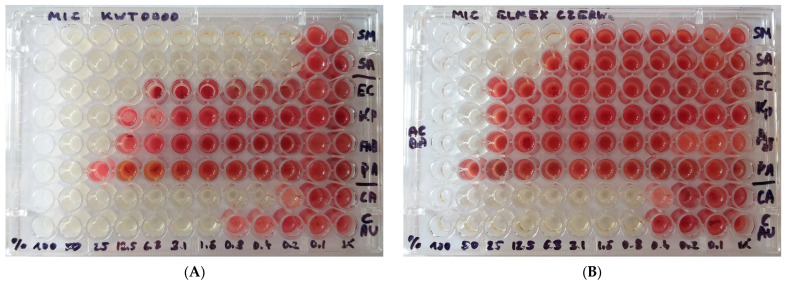
Example images showing the effect of (**A**). KWT0000 and (**B**). ELM on selected strains. The absence of growth is visible in selected wells at KWT0000 concentrations ranging from 0.2% to 50% and at ELM concentrations ranging from 0.8% to 100%. Strain abbreviations: SM—*Streptococcus mutans*, SA—*Staphylococcus aureus*, EC—*Escherichia coli*, KP—*Klebsiella pneumoniae*, AB—*Acinetobacter baumannii*, PA—*Pseudomonas aeruginosa*, CA—*Candida albicans*, CAU—*C. auris*.

The studies demonstrated very good activity of KWT0000 against Gram-positive bacteria and *Candida* fungi, with MICs between 0.2% and 1.6%. Simultaneously, the solution had moderate activity against Gram-negative bacteria, with MICs between 12.5% and 50%. At the same time, ELM exhibited similar activity against Gram-negative bacteria and *Candida* fungi, with MICs of 50–100% and 0.4–1.8%, respectively. This mouthwash showed considerably weaker activity against Gram-positive bacteria, with MICs ranging from 6.3% to 12.5% (Figure 1).

**Figure 2:**
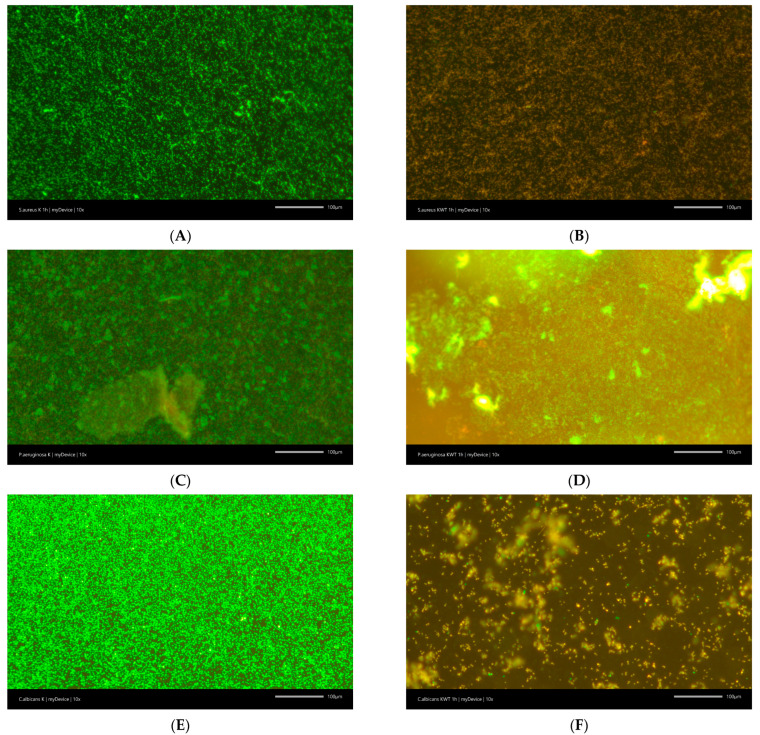
Example images demonstrating the reduction of biofilm under the influence of KWT0000: (**A**)—Control sample for *S. aureus*, (**B**)—Sample with KWT0000 after 1 h for *S. aureus*, (**C**)—Control sample for *P. aeruginosa*, (**D**)—Sample with KWT0000 after 1 h for *P. aeruginosa*, (**E**)—Control sample for *C. albicans*, (**F**)—Sample with KWT0000 after 1 h for *C. albicans*.

KWT0000 and ELM also exhibit anti-biofilm activity, leading to the killing of *S. aureus* and *C. albicans* cells in as little as 1 h. KWT0000 exhibited slightly stronger anti-biofilm activity and a greater reduction in cell viability compared to ELM. Moreover, KWT0000 demonstrated weak anti-biofilm activity against *S. aureus*, which was not observed for ELM, despite a statistically significant decrease in the viability of bacterial cells. In contrast, against *P. aeruginosa*, these agents failed to exert a significant anti-biofilm effect, as most bacterial cells remained viable and biofilm integrity was preserved (Table 1, Figure 2).

After just one hour of exposure to KWT0000, a significant reduction in the number of viable *S. aureus* cells within biofilms was observed under fluorescence microscopy. Compared to the untreated control samples, which exhibited dense and metabolically active biofilms, treated samples showed a predominance of dead cells, indicating effective penetration and antimicrobial action of the mouthwash within the biofilm matrix. This is particularly relevant, as biofilm-embedded bacteria are often far more resistant to antimicrobial agents than their planktonic counterparts.

Then, the antibiofilm activity against *S. aureus* was evaluated to assess the potential of the tested compound in preventing or disrupting biofilm formation on different types of dental materials. The selection of materials such as poly (methyl methacrylate) (PMMA), resin A1, anodized titanium, polished titanium, zirconium solo, zirconium glaze, and polished zirconia represents a strategic choice based on their widespread use, diverse surface properties, and relevance to clinical dental applications. These materials are commonly encountered in fixed and removable prosthodontics, implantology, and restorative dentistry, making them highly representative of the oral environment. PMMA and resin A1 are frequently used in temporary restorations and removable prostheses due to their ease of manipulation and acceptable esthetics. Titanium, in both anodized and polished forms, is a gold standard material for dental implants and abutments due to its excellent biocompatibility and mechanical strength, with surface treatments such as anodization potentially influencing bacterial adhesion and tissue integration. Zirconia, in its various surface finishes—solo (monolithic), glazed, and polished—is widely used in crowns, bridges, and implant-supported restorations for its superior mechanical properties, esthetics, and favorable tissue response. Studying this selection allows for the evaluation of microbial adhesion and material performance across a spectrum of clinically relevant surfaces, ranging from polymers to metals to ceramics, each with distinct topographical and chemical characteristics. This provides comprehensive insight into how various restorative and prosthetic materials may influence microbial colonization and oral health outcomes.

**Figure 3:**
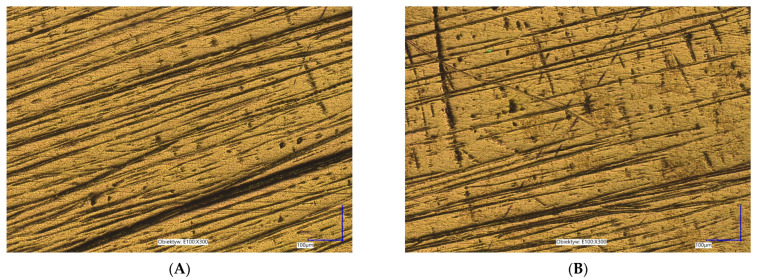

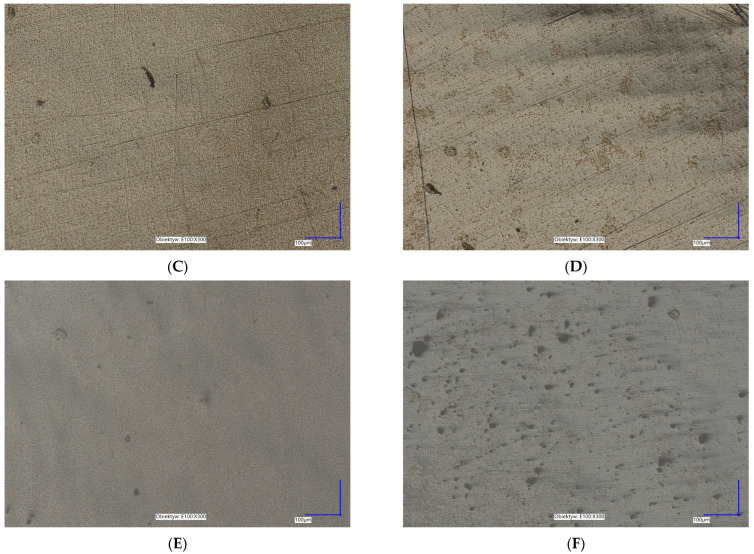
Sample images showing the effect of KTW0000 on *Staphylococcus aureus* biofilm reduction on various dental materials: (**A**)—Anodized titanium Control; (**B**)—Anodized titanium with KWT0000 after 1 h; (**C**)—Polished titanium Control; (**D**)—Polished titanium with KWT0000 after 1 h; (**E**)—Polished zirconia Control; (**F**)—Polished zirconia with KWT0000 after 1 h.

It was found that KWT0000 reduced the number of bacterial cells on the surface of the tested materials to varying degrees, depending on the structure of the material (Figure 3).

On polished titanium and polished zirconia surfaces, a pronounced decrease in bacterial coverage was visible after treatment, suggesting that smoother and less porous surfaces allow better interaction of the active compounds with the biofilm. In contrast, on PMMA and glazed zirconia surfaces, the biofilm persisted to a greater extent, with bacteria still widely distributed after one hour of treatment. These differences may be attributed to variations in surface free energy, roughness, and material-specific interactions with bacterial adhesins, which can modulate the adherence and resistance of biofilms.

Anodized titanium also showed relatively low biofilm density even in the untreated state, which may indicate inherent antiadhesive properties of this surface. The observation that KWT0000 further reduced bacterial presence on this material suggests a potential synergistic benefit when using this mouthwash in patients with anodized implant components.

The rapid onset of action, visible within just one hour, underscores the potential clinical relevance of KWT0000 in peri-implant maintenance and in early intervention settings where bacterial biofilm control is critical. Given that *S. aureus* is a common pathogen involved in peri-implantitis and soft tissue infections, an agent capable of rapid biofilm disruption is of high therapeutic value.

Taken together, these results suggest that KWT0000 not only possesses antimicrobial properties in planktonic culture (as confirmed by MIC values) but also exerts meaningful effects on mature biofilms of clinically relevant pathogens. The formulation’s efficacy appears to be enhanced on metallic surfaces such as titanium, which are commonly used in dental implants. Further studies may be warranted to optimize formulation contact time and delivery methods, especially on more biofilm-retentive surfaces such as PMMA or glazed ceramics.

The observed antibiofilm activity of the nano-hydroxyapatite-based mouthwash (KWT0000) against *S. aureus* is consistent with findings reported in recent literature, which highlight the potential of nano-hydroxyapatite (nanoHAP) as a bioactive agent capable of interfering with microbial adhesion and biofilm formation.

Several studies have demonstrated that nanoHAP can reduce bacterial colonization by modifying surface properties and interfering with bacterial adhesion mechanisms. NanoHAP particles adsorb onto tooth and implant surfaces, creating a bioactive layer that inhibits bacterial attachment and supports tissue remineralization [24]. This biofilm-modulating effect is particularly relevant in early-stage colonization by Gram-positive organisms such as *S. aureus*, which rely on specific adhesins to bind to host and biomaterial surfaces [25].

Additionally, hydroxyapatite’s capacity to chelate metal ions and its surface charge may contribute to biofilm destabilization [26]. Some reports propose that nanoHAP’s nanoscale morphology and high surface area enhance its interaction with bacterial membranes, potentially leading to mechanical disruption or interference with nutrient transport within biofilms [27].

Specifically for *S. aureus*, recent in vitro studies have shown that nanoHAP-based formulations can decrease both adhesion and biofilm biomass. H-coated surfaces showed significantly lower *S. aureus* biofilm formation compared to uncoated controls [28]. Similarly, nanoHAP can disrupt mature *S. aureus* biofilms, especially when combined with antimicrobial agents such as zinc or CPC [29], both of which are present in the KWT0000 formulation.

This synergistic potential is crucial: hydroxyapatite may act as a carrier or enhancer for other bioactive molecules, facilitating their penetration into the biofilm structure. In the case of KWT0000, the combination of nanoHAP with zinc lactate, D-panthenol, licorice extract, and cetylpyridinium chloride likely results in a multifaceted mode of action, combining physical interference with bacterial adhesion, chemical antimicrobial effects, and anti-inflammatory benefits.

Therefore, the results presented in our study not only align with existing evidence on the antibiofilm properties of nanoHAP but also suggest that its efficacy can be significantly enhanced through well-designed formulation strategies. These findings reinforce the potential of nanoHAP-containing products in the prevention and management of biofilm-related oral pathologies, particularly in vulnerable areas such as peri-implant tissues.

The analysis of antioxidant properties revealed that the KWT0000 mouthwash demonstrated significantly higher radical scavenging activity (55.33 ± 3.34%) compared to the comparative mouthwash (17.51 ± 1.55%) (Table 2). Similarly, in the assessment of anti-inflammatory potential via hyaluronidase inhibition, KWT0000 exhibited a markedly greater activity (23.33 ± 3.67%) than the comparative product (3.67 ± 0.26%).

The significantly higher antioxidant and anti-inflammatory activities observed for the KWT0000 mouthwash can be directly linked to the synergistic effects of its bioactive ingredients: hydroxyapatite, licorice extract, D-panthenol, zinc lactate, and cetylpyridinium chloride. Licorice extract is a key contributor to both antioxidant and anti-inflammatory effects. It contains a variety of bioactive compounds such as flavonoids (e.g., liquiritin, isoliquiritigenin) and glycyrrhizin. Flavonoids in licorice are potent free radical scavengers; they can neutralize reactive oxygen species (ROS), thus protecting cells from oxidative damage [30]. Glycyrrhizin inhibits the release of pro-inflammatory cytokines (such as TNF-α and IL-6) and suppresses the activation of NF-κB, a key transcription factor involved in inflammatory responses [31]. D-Panthenol (provitamin B5) plays a supportive role in tissue repair and inflammation control. D-Panthenol can scavenge ROS and reduce lipid peroxidation, thereby stabilizing cellular membranes. It enhances skin and mucosal barrier function and reduces irritation and inflammation by promoting fibroblast proliferation and tissue regeneration [32]. Zinc acts as a cofactor for superoxide dismutase (SOD), one of the major antioxidant enzymes in the body, helping to neutralize superoxide radicals. Zinc modulates the immune response by reducing the production of pro-inflammatory cytokines and stabilizing cell membranes against oxidative insults [33]. Although hydroxyapatite is primarily known for its remineralization properties, it may also indirectly support mucosal tissue health. It helps to maintain the integrity of oral surfaces, reducing tissue vulnerability to oxidative and inflammatory damage triggered by bacterial plaque and environmental stressors [34]. Additionally, cetylpyridinium chloride (CPC) is mainly known for its antimicrobial properties, but by effectively reducing the bacterial load in the oral cavity, CPC lowers the amount of bacterial endotoxins (such as lipopolysaccharides) that can trigger inflammatory responses [5].

In contrast, the comparative mouthwash mainly contains Olaflur and sodium fluoride, ingredients primarily aimed at enhancing enamel remineralization and caries prevention rather than providing significant antioxidant or anti-inflammatory effects. The sodium salt of anise acid present in the comparative product may offer mild biological activity; however, it does not match the potency of licorice extract or zinc-related compounds found in KWT0000.

Thus, the superior antioxidant and anti-inflammatory activities observed for KWT0000 are consistent with the bioactive profiles of its specific ingredients, highlighting its potential not only in oral hygiene maintenance but also in supporting soft tissue health.

While the present study provides robust evidence for the antimicrobial and anti-biofilm efficacy of the nano-hydroxyapatite-based mouthwash (KWT0000), it is important to contextualize these findings within the broader landscape of mouthwash formulations evaluated in previous studies. Chlorhexidine gluconate (CHX), widely regarded as the “gold standard” in oral antiseptics, has consistently shown strong activity against *Streptococcus mutans*, *Candida albicans*, and other oral pathogens [35]. Its mechanism involves membrane disruption and precipitation of cytoplasmic contents [36]. However, its long-term use is associated with significant adverse effects, including tooth discoloration, altered taste perception, and mucosal desquamation. These side effects limit its use as a long-term preventive agent, particularly in patients with mucosal sensitivity or implant-retained prostheses [37]. On the other hand, fluoride-based mouthwashes, especially those containing amine fluoride or stannous fluoride, have demonstrated efficacy in reducing demineralization and controlling gingival inflammation [38]. Clinical trials showed that stannous fluoride formulations reduced plaque accumulation and bleeding on probing more effectively than sodium fluoride alone [39]. However, their bactericidal activity is typically limited to specific pathogens, and they show relatively poor performance against multidrug-resistant organisms such as *Acinetobacter baumannii* and *Pseudomonas aeruginosa*, which are increasingly relevant in immunocompromised or hospitalized patients [40]. Essential oil-based mouthwashes (e.g., Listerine) are known for their broad-spectrum antimicrobial effects, primarily through disruption of bacterial cell walls [41]. While effective against common oral flora, these formulations often include ethanol as a solvent, which may cause mucosal irritation and is contraindicated in certain patient populations. Moreover, their efficacy in disrupting mature biofilms or preventing recolonization on implant surfaces remains inferior compared to CHX or more advanced biomimetic agents. Mouthwashes containing cetylpyridinium chloride (CPC), a quaternary ammonium compound also included in KWT0000, exhibit bacteriostatic and bactericidal properties with reduced side effects compared to CHX [5]. However, standalone CPC mouthwashes may have limited anti-inflammatory properties and are less effective against fungi such as *Candida* spp. [42]. The inclusion of CPC in synergy with nano-hydroxyapatite, as seen in KWT0000, likely enhances both its efficacy and tolerability. Recent research has turned toward more biocompatible and multifunctional formulations. For instance, probiotics and postbiotic-enriched mouthwashes have been explored for oral microbiota modulation [43], but their clinical efficacy remains under investigation. Similarly, green tea polyphenol-based rinses and herbal formulations (e.g., containing *Salvadora persica*, *Curcuma longa*, or *Camellia sinensis*) have shown anti-inflammatory and antimicrobial effects, though their activity is often weaker or more variable depending on concentration and formulation stability [44].

In comparison to these existing mouthwash types, the nano-hydroxyapatite-based KWT0000 formulation offers several advantages. In our study, it exhibited comparable or superior antibiofilm efficacy to conventional agents, especially on titanium and zirconia surfaces, materials commonly used in implantology. Furthermore, the incorporation of antioxidant (e.g., D-panthenol, licorice extract) and anti-inflammatory components enhances its therapeutic value beyond mechanical biofilm disruption. Notably, nano-hydroxyapatite has also been associated with remineralization properties and improved mucosal compatibility [34], making it a favorable alternative for long-term use. Taken together, these findings suggest that nano-hydroxyapatite-based formulations such as KWT0000 may fill a critical gap in oral care, providing effective antimicrobial and anti-biofilm action without the side effects commonly seen in traditional antiseptics. This is particularly important for patients with dental implants, peri-implant mucositis, or oral soft tissue sensitivity. Future studies directly comparing KWT0000 with CHX, fluoride-based, and herbal rinses in clinical settings will be essential to further establish its position within preventive dentistry protocols.

Despite the promising results obtained, this study has certain limitations that should be acknowledged when interpreting the findings. Firstly, the experiments were conducted under in vitro conditions, which, although controlled and reproducible, do not fully replicate the complexity of the oral environment in vivo. Laboratory-grown biofilms lack the structural diversity, microbial variability, and protective mechanisms present in naturally formed oral biofilms. Moreover, the study focused on selected reference and clinical strains, which may not represent the full spectrum of oral microbiota observed in diverse patient populations. Another limitation is the single exposure time of one hour used in evaluating the mouthwashes. This does not reflect the dynamic nature of biofilm formation over time or the potential cumulative effects of long-term product use. The study also did not account for the influence of saliva, enzymes, or interactions with other substances in the oral cavity that may affect the product’s efficacy.

From a developmental perspective, future research should include both short- and long-term in vivo clinical trials involving patients with natural dentition, prosthetic restorations, and dental implants. Of particular interest are studies evaluating the product’s effectiveness in preventing peri-implantitis, reducing plaque accumulation, improving soft tissue health, and confirming safety during prolonged daily use.

Overall, the tested formulation appears to be a promising multifunctional oral care product, and its further development and clinical validation are highly warranted.

## 4. Conclusions

This study demonstrated that the nano-hydroxyapatite-based mouthwash (KWT0000) exhibits potent antimicrobial and antibiofilm activity, particularly against Staphylococcus aureus and Candida albicans. Notably, the formulation significantly reduced viable biofilm cells within one hour of exposure, with enhanced effectiveness on metallic surfaces, such as polished and anodized titanium.

The findings highlight the potential of KWT0000 in preventing peri-implant diseases by disrupting early biofilm formation, a key factor in implant failure. The synergistic effects of nano-hydroxyapatite with zinc lactate, cetylpyridinium chloride, D-panthenol, and licorice extract further contribute anti-inflammatory and antioxidant benefits, enhancing its therapeutic profile.

Given its rapid action and multifunctional properties, KWT0000 represents a promising adjunct to oral hygiene regimens, particularly for individuals with dental implants or prosthetic restorations. Future in vivo studies and clinical trials are needed to validate its long-term efficacy and safety in daily use.

## Figures and Tables

**Table 1:** Reduction of biofilm biomass and changes in cell viability under the influence of KWT0000 and ELM.

Mouthwash	Antibiofilm Activity	Pathogens		
		*Staphylococcus aureus*	*Pseudomonas aeruginosa*	*Candida albicans*
Control	Biofilm %	100	100	100
	Viability %	98.3 ± 1.5	94.0 ± 1.0	98.3 ± 0.6
KWT0000	Biofilm reduction %	−7.7 ± 2.5 *	−4.6 ± 2.5	−64.7 ± 5.0 ***
	Viability %	31.7 ± 5.8 ***	95.3 ± 1.2	40.0 ± 5.0 ***
ELM	Biofilm reduction %	−6.3 ± 1.5	−4.7 ± 2.5	−46.7 ± 7.6 ***
	Viability %	38.3 ± 2.9 ***	94.7 ± 1.2	50.0 ± 5.0 ***

Statistical significance compared to the control group: *—*p* < 0.05, ***—*p* < 0.001.

**Table 2:** Antioxidant and anti-inflammatory properties of tested mouthwashes.

	Antioxidant Properties [%]	Anti-Inflammatory Properties [%]
KWT0000	55.33 ± 3.34 ^a^	23.33 ± 3.67 ^a^
ELM mouthwash	17.51 ± 1.55 ^b^	3.67 ± 0.26 ^b^

Mean values in a column marked with the same letter are not statistically significantly different at *p* = 0.05, according to Duncan’s test.

## Data Availability

The original contributions presented in this study are included in the article. Further inquiries can be directed to the corresponding author.
